# The Effect of Flywheel Inertia on Peak Power and Its Inter-session Reliability During Two Unilateral Hamstring Exercises: Leg Curl and Hip Extension

**DOI:** 10.3389/fspor.2022.898649

**Published:** 2022-06-10

**Authors:** Kevin L. de Keijzer, Stuart A. McErlain-Naylor, Marco Beato

**Affiliations:** ^1^School of Health and Sport Sciences, University of Suffolk, Ipswich, United Kingdom; ^2^Institute of Health and Wellbeing, University of Suffolk, Ipswich, United Kingdom; ^3^School of Sport, Exercise and Health Sciences, Loughborough, United Kingdom

**Keywords:** inertia, power, training, flywheel, hamstrings

## Abstract

This study investigated the effect of flywheel moment of inertia (0.029, 0.061, and 0.089 kg·m^2^) on concentric and eccentric peak power and eccentric:concentric peak power ratio during unilateral flywheel leg curl and hip extension exercises. Moreover, the inter-session reliability of peak power was analyzed during both exercises. Twenty amateur male soccer athletes attended five visits—performing three sets of eight repetitions of either unilateral leg curl or hip extension (all three moments of inertias) during each visit. For the unilateral leg curl, there were no differences in any measure between moments of inertia (*p* = 0.479) but a higher eccentric than concentric peak power for all moments of inertia (*p* < 0.001). For the unilateral hip extension, differences between moments of inertia were reported for all measures (*p* < 0.05). Specifically, the lowest moment of inertia elicited the greatest concentric peak power (*p* = 0.022), there were no differences with the medium inertia (*p* = 0.391), and the greatest moment of inertia obtained the greatest eccentric peak power (*p* = 0.036). Peak power measures obtained acceptable to excellent reliability while the eccentric:concentric ratio reported unacceptable to good reliability for both exercises. A variety of moments of inertia can elicit high eccentric knee flexor demands during unilateral leg curls, whereas higher moments of inertia are needed to achieve an eccentric-overload in peak power during hip extensions. Different exercises may have different inertia-power relationships. Concentric and eccentric peak power measures should continue to inform training, while the eccentric:concentric ratio should not be used.

## Introduction

The physical demands of soccer necessitate effective strength and conditioning programs to optimize long-term development of physical capacities and reduce likelihood of injury (Beato et al., 2018, 2020). The physical preparation and training “load” management of elite soccer players remains a complex challenge during congested fixture periods and throughout the competitive season (Gualtieri et al., 2020; de Keijzer et al., 2022). This challenge is augmented by high incidence and recurrence of muscular injuries, particularly hamstring strain injuries (Beato et al., 2020). In an attempt to reduce injury likelihood and improve performance with soccer players, a variety of training methods have been implemented with a particular focus on eccentric based exercises (Askling et al., 2003; Fernandez-Gonzalo et al., 2016; Coratella et al., 2019; Allen et al., 2021). Although such training provides unique neuromuscular benefits (Norrbrand et al., 2010; Tesch et al., 2017), it remains difficult to program with athletic populations (Harden et al., 2020).

Flywheel training is a training modality that has been effectively implemented to enhance strength and key performance parameters within sporting populations (Raya-González et al., 2021; de Keijzer et al., 2022). Specifically, flywheel training can enhance change of direction (COD), jump performance and strength parameters of male soccer players (Allen et al., 2021). Movements using the flywheel ergometer are initiated by a concentric phase that rotates the flywheel disc(s) and thereby generates inertial torque. Upon completing the pre-determined range of motion, the flywheel disc(s) continue to rotate, and the inertial torque provides resistance, requiring the user to decelerate the disc(s) during the eccentric phase of the movement. The flywheel device enables near-maximal effort in both concentric and eccentric phases of each repetition and possibly an eccentric overload (Norrbrand et al., 2011; Tesch et al., 2017)—where eccentric output exceeds the concentric output (Muñoz-López et al., 2021a). Although the ability to achieve an eccentric overload is considered important to practitioners working in soccer (de Keijzer et al., 2022), it is not consistently attained in the literature and may be largely dictated by moment of inertia, angular velocity, device type and exercise selected (Muñoz-López et al., 2021a).

Guidelines for implementation and management of flywheel training in soccer are currently lacking (Beato and Dello Iacono, 2020; Beato et al., 2020), possibly limiting its systematic implementation (Sabido et al., 2018; Muñoz-López et al., 2021a). Although the use of arbitrary moments of inertia are utilized with flywheel devices (Lundberg et al., 2019; Presland et al., 2020), objective quantification to enhance training has become of increasing interest and practicality (Maroto-Izquierdo et al., 2021; Muñoz-López et al., 2021a). Specifically, the measurement of concentric and eccentric peak power (and the eccentric:concentric peak power ratio) have become more frequently utilized (Muñoz-López et al., 2021a). The literature has attempted to quantify how moments of inertia impact flywheel resistance training measures (such as peak power) to improve training outcomes and load management (Sabido et al., 2018; Muñoz-López et al., 2021b; O'Brien et al., 2021; Puustinen et al., 2021). Altering inertia (analyzed at group level rather than individually) did not affect peak power or trunk lean, but did alter velocity—highlighting that not all training variables may be equally effective for determining training intensity (McErlain-Naylor and Beato, 2020; Worcester et al., 2020). It also remains unclear which kinetic or kinematic variables can be used to determine intensity for various flywheel exercises. In theory, the intramuscular force-velocity-power relationships likely lead to force and velocity being maximized at greater and lower external resistance, respectively (Hill, 1938). Therefore, power (i.e., force × velocity) should theoretically be maximized at an intermediate resistance (Hill, 1938). Although technological advances and improvements in the ability to quantify training have been made—many questions remain (Maroto-Izquierdo et al., 2021; Muñoz-López et al., 2021a). For example, various factors (e.g., participant characteristics, exercise, and inertia) may place the individual on the ascending or descending portion of their specific inertia-power relationship, highlighting that arbitrary and pre-determined changes in inertia may result in unknown peak power outcomes (McErlain-Naylor and Beato, 2020).

Unilateral knee extensions utilizing higher rather than lower moments of inertia have resulted in greater concentric and eccentric peak power values (Martinez-Aranda and Fernandez-Gonzalo, 2017). In contrast, other exercises (e.g., squats, deadlifts) reported higher peak power at lower, rather than higher, moments of inertias (Sabido et al., 2018; Piqueras-Sanchiz et al., 2020; O'Brien et al., 2021). Furthermore, eccentric overload was achieved only with higher moments of inertia in certain investigations (Sabido et al., 2018; O'Brien et al., 2021), whereas other exercises achieved eccentric overload regardless of the moment of inertia used (Martinez-Aranda and Fernandez-Gonzalo, 2017; Piqueras-Sanchiz et al., 2020). Eccentric overload and peak power values may therefore depend upon execution and exercise selection. It is of interest to see whether the leg curl and hip extension exercises (which elicit different activation patterns and occupy different portions of the hamstrings force-length curve) (Ono et al., 2010; Presland et al., 2020) uniformly respond to changes in moments of inertia. No previous investigation has determined how moment of inertia affects peak power output during unilateral leg curl. Similarly, it remains unclear whether altering moment of inertia during unilateral hip extension affects peak power and elicits an eccentric overload (Piqueras-Sanchiz et al., 2019). Further investigation into the effect of manipulating key exercise parameters during unilateral flywheel hamstring exercises are necessary for improving flywheel training management and prescription (Beato and Dello Iacono, 2020; Beato et al., 2020).

The aim of this study was to investigate the effect of flywheel moment of inertia (0.029, 0.061, and 0.089 kg·m^2^) on concentric and eccentric peak power and eccentric:concentric peak power ratio during unilateral flywheel leg curl and hip extension exercises. Moreover, a second aim was to analyze the inter-session reliability of the concentric and eccentric peak power and eccentric:concentric peak power ratio during both exercises. It was hypothesized that lower inertias would result in greater peak power values and that eccentric overload will only be attained with higher moments of inertia, as seen with bilateral and unilateral flywheel exercises (Martinez-Aranda and Fernandez-Gonzalo, 2017; Sabido et al., 2018; O'Brien et al., 2021).

## Methods

### Experimental Approach to the Problem

**Figure 1:**
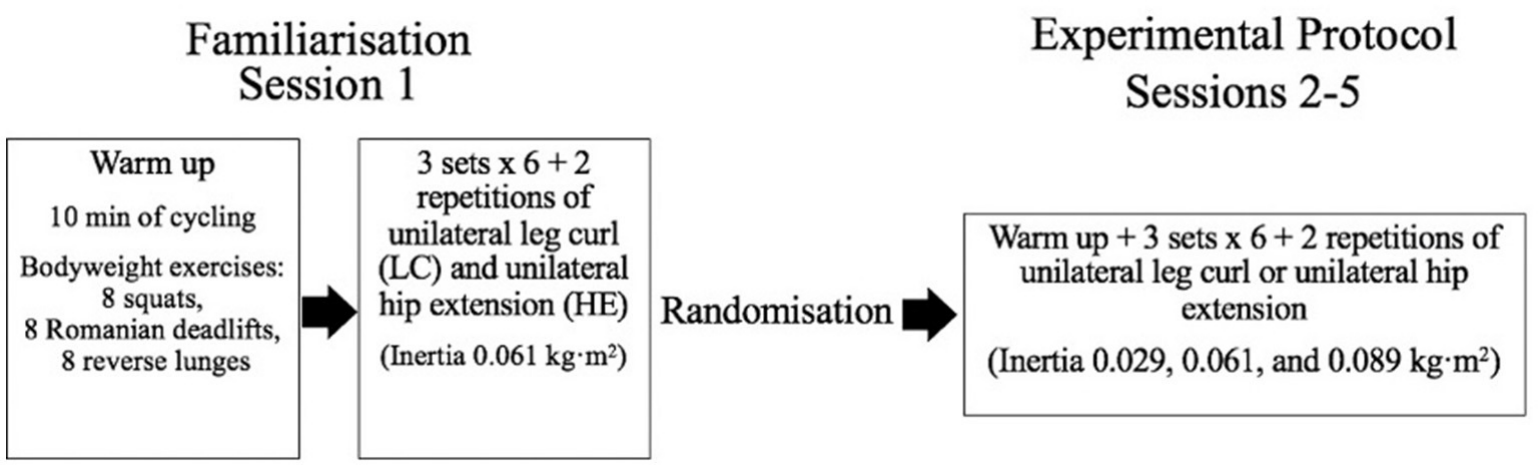
Experimental design. Min = minutes. 6 + 2 repetitions = 6 maximal repetitions with 2 initial repetitions utilized to initiate the movement.

The study utilized a cross-sectional design to determine the impact of different flywheel moments of inertia during unilateral flywheel leg curl and hip extension on concentric and eccentric peak power and eccentric:concentric peak power ratio. Each participant attended the laboratory five times over a 3-week period, with the study design presented in Figure 1. An initial familiarization session was performed, with both limbs performing the unilateral leg curl and unilateral hip extension exercises. In the subsequent sessions, participants were randomized to perform one exercise (leg curl or hip extension) per session with their self-selected kicking leg, using all moments of inertia in each session (Figure 1). The randomization of order of moment of inertia and a 2 min rest period between sets were prescribed to minimize the effects of accumulated fatigue on performance. The testing procedures were repeated with the same methodology to enable an analysis of the inter-session reliability for both exercises (performed in the subsequent week).

### Subjects

Twenty healthy male university soccer athletes (age 22 ± 2 years, height 1.82 ± 0.04 m, body mass 83.4 ± 8.8 kg) voluntarily participated in this investigation. An a *priori* power analysis was conducted to determine the appropriate sample size using G^*^Power (version 3.1.9.3, Düsseldorf, Germany). Considering the study design (1 group, 2 repeated measures), a medium effect size f = 0.3, a correlation between measurements of *r* = 0.6, an α = 0.05, a required power 1-β = 0.80, a non-sphericity correction ε = 1, a sample of 20 participants was required (actual power = 0.81). The participants were investigated during the off-season period to avoid interfering with team-based training and competition. All participants were informed of the risks associated with the study procedures. Prior to participating, every participant signed written informed consent approved by the research ethics committee of the university of x in accordance with the Declaration of Helsinki.

### Procedures

Body mass and height were recorded by stadiometer (Seca 286dp; Seca, Hamburg, Germany). A standardized warm-up consisting of 10 min of cycling at 80–100 W (Wattbike, Nottingham, UK) and bodyweight lower body movements were performed prior to every session (Figure 1). Participants were requested not to take anti-inflammatory or additional supplementation (i.e., caffeine) on the day of testing. In the 48 h preceding testing protocols, participants were asked to avoid strenuous exercise. Testing occurred at similar times of the day with participants asked to maintain habitual diet and fluid intake for the duration of the study.

#### Leg Curl and Hip Extension

Range of motion for the leg curl exercise was determined by the researcher prior to each set, ranging from approximately a 30° knee flexion angle (start of concentric/end of eccentric) to a 120° knee flexion angle in the sagittal plane (end of concentric/start of eccentric). Similarly, hip extension (measured in anatomical position) range of motion was from ~90° hip flexion/extension angle (start of concentric/end of eccentric) and ended at 0–5° of hip extension angle in the sagittal plane (end of concentric/start of eccentric). A manual goniometer was used to set the initial range of motion, which was subsequently assessed qualitatively by the same researcher during familiarization and testing for both exercises. The exercises can be viewed in Supplementary Figures 1, 2. Moments of inertia utilized in this study were 0.029, 0.061, and 0.089 kg·m^2^, based on the wide range of moments of inertia previously investigated (Piqueras-Sanchiz et al., 2020). The investigated moments of inertia utilized different (flywheel) discs and are specified here: 0.029 kg·m^2^ [1 large disc (diameter = 0.285 m; mass = 1.9 kg; inertia = 0.02 kg·m^2^) and 1 medium disc (diameter = 0.240 m; mass = 1.1 kg; inertia = 0.008 kg·m^2^)]; 0.061 kg·m^2^ [1 pro disc (diameter = 0.285 m; mass = 6.0 kg; inertia = 0.060 kg·m^2^)]; and 0.089 kg·m^2^ (1 pro, 1 large, and 1 medium disc). The inertia of the ergometer was estimated as 0.0011 kg·m^2^ and has already been considered in the reported moments of inertia (Figure 1).

Participants were encouraged to perform the concentric phase as quickly as possible and delay the braking phase of the eccentric portion as much as possible to enhance the eccentric phase (Tesch et al., 2017). Concentric and eccentric peak power were recorded *via* an in-built rotational encoder (V11Full, Desmotec, Biella, Italy) (Beato et al., 2019). The peak power of 3 sets was averaged over all experimental sessions (sessions 2–5) and was subsequently analyzed.

### Statistical Analysis

All statistical analyses were performed using JASP software version 0.13.1 for Mac (Amsterdam, Netherlands). All data were assessed for normality using the Shapiro-Wilk test and are presented as mean ± standard deviation (SD). Repeated measures analysis of variance (ANOVA) were employed to measure potential within condition (flywheel moment of inertia) differences for the unilateral leg curl and hip extension. A sphericity assumption check was performed using Mauchly's test, and if a violation was found (*p* > 0.05), the Greenhouse-Geisser correction was applied. Statistical significance was set at *p* < 0.05. When significant F-values were reported, *post-hoc* analyses were performed (with Bonferroni correction applied to the alpha value). Paired-samples *t*-tests were applied to analyze differences between concentric and eccentric peak power outcomes. Robust estimates of 95% confidence intervals (CI) and heteroscedasticity were calculated using the bootstrapping technique (1,000 randomly bootstrapped samples). Effect size based on the Cohen's *d* principle was interpreted as: *trivial* < 0.2; 0.2 ≤ *small* < 0.6; 0.6 ≤ *moderate* < 1.2; 1.2 ≤ *large* < 2.0; *very large* ≥ 2.0 (Hopkins et al., 2009). The reliability of the measures was assessed through a two-way mixed model intraclass correlation coefficient (ICC) and interpreted as: *excellent* > 0.9; 0.9 ≥ *good* > 0.8; 0.8 ≥ *acceptable* > 0.7; 0.7 ≥ *questionable* > 0.6; 0.6 ≥ *poor* > 0.5; and *unacceptable* < 0.5 (Atkinson and Nevill, 1998).

## Results

### Unilateral Flywheel Leg Curl

**Figure 2:**
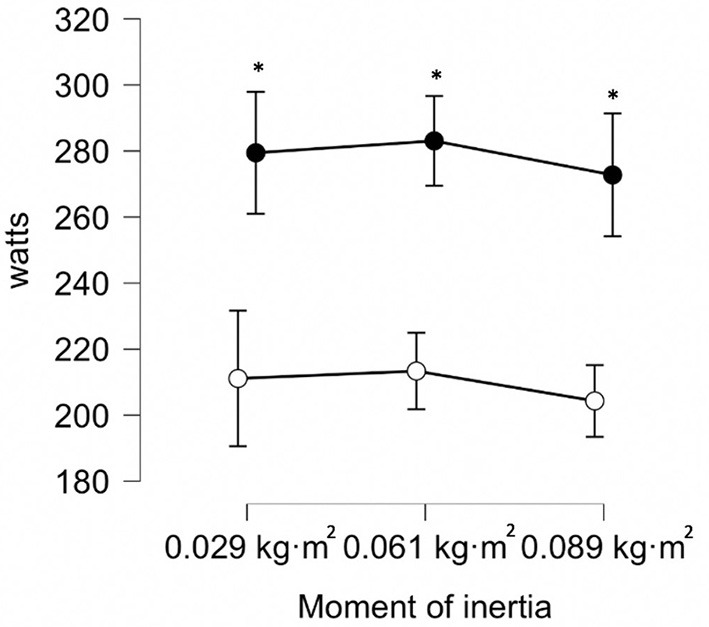
Concentric and eccentric peak power during unilateral flywheel knee flexion. *Statistically significant (*p* < 0.05) difference between concentric and eccentric peak power. Black dots = eccentric peak power values. White dots = concentric peak power values.

All data were normally distributed (*p* > 0.05). Repeated measures ANOVAs detected no difference in peak power between moments of inertia for concentric (*F* = 0.62; *p* = 0.479), eccentric (*F* = 0.50; *p* = 0.564), or eccentric:concentric ratio (*F* = 0.07; *p* = 0.934), therefore a *post-hoc* analysis was not performed. Significant differences (*p* < 0.001) between eccentric peak power and concentric peak power measures were reported for all moments of inertia (Figure 2). Specifically, differences between concentric and eccentric peak power (greater eccentric power) were found at 0.029 kg·m^2^ [68 W (47–90); *d* = 1.46 (0.82–2.09)], 0.061 kg·m^2^ [70 W (53–87); *d* = 1.92 (1.61–2.66)], and 0.089 kg·m^2^ [69 W (49–88); *d* = 1.68 (0.98–2.36)].

### Unilateral Flywheel Hip Extension

The repeated measures ANOVA detected an effect between moments of inertia for concentric peak power (*F* = 9.07; *p* = 0.002), eccentric peak power (*F* = 16.85; *p* < 0.001), and eccentric:concentric ratio (*F* = 14.17; *p* < 0.001). *Post-hoc* results are presented in Table 1.

**Table 1:** Flywheel unilateral hip extension *post-hoc* tests for the repeated measures ANOVAs.

**Moment of inertia**		**Watts** **mean difference** **(95% CI)**	**Cohen's *d*** **(*Interpretation*)**	***p-*** **value**
**Concentric**				
0.029 kg·m^2^ vs	0.061 kg·m^2^	15 (5–35)	0.42 (*small*)	0.207
	0.089 kg·m^2^	34 (14–54)	0.95 (*moderate*)	<0.001^*^
0.061 kg·m^2^ vs	0.089 kg·m^2^	19 (1–39)	0.53 (*small*)	0.068
**Eccentric**				
0.029 kg·m^2^ vs	0.061 kg·m^2^	20 (7–46)	0.42 (*small*)	0.204
	0.089 kg·m^2^	60 (34–87)	1.27 (*large*)	<0.001^*^
0.061 kg·m^2^ vs	0.089 kg·m^2^	40 (14–67)	0.85 (*moderate*)	0.001^*^
**E:C ratio**				
0.029 kg·m^2^ vs	0.061 kg·m^2^	0.01 (0.02–0.04)	0.16 (*trivial*)	1.00
	0.089 kg·m^2^	0.06 (0.03–0.08)	1.10 (*moderate*)	<0.001^*^
0.061 kg·m^2^ vs	0.089 kg·m^2^	0.05 (0.02–0.07)	0.94 (*moderate*)	<0.001^*^

*CI, Confidence intervals;*

* *p < 0.05*.

**Figure 3:**
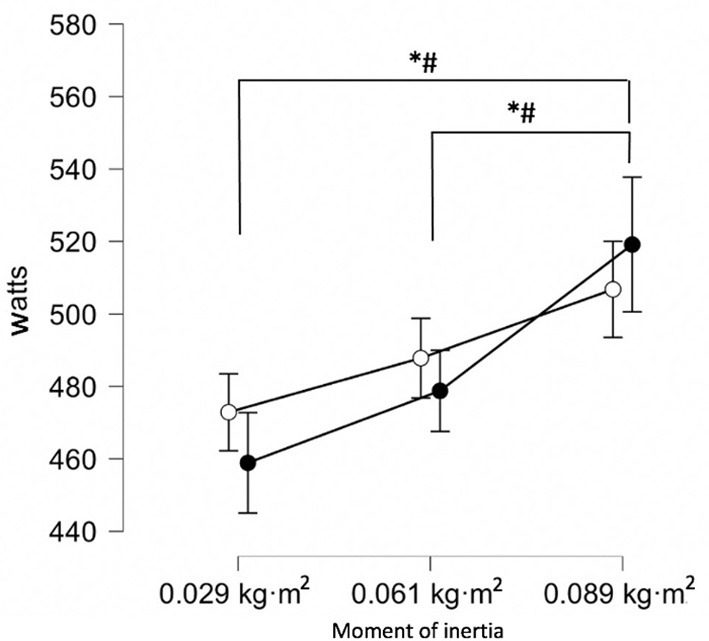
Concentric and eccentric peak power output during unilateral flywheel hip extension. *Statistically significant (*p* < 0.05) difference between concentric peak power. ^#^Statistically significant (*p* < 0.05) difference between eccentric peak power. Black dots = eccentric peak power output. White dots = concentric peak power output.

Significant differences between concentric and eccentric peak power were reported for 0.029 kg·m^2^ [*p* = 0.022; 14 W (2–26); *d* = 0.56 (0.08–1.03)] and 0.089 kg·m^2^ [*p* = 0.036; 12 W (1–23); *d* = 0.50 (0.31–0.96)], but not for 0.061 kg·m^2^ [*p* = 0.391; 9 W (2–20); *d* = 0.39 (0.07–0.84)], as presented in Figure 3.

**Figure 4:**
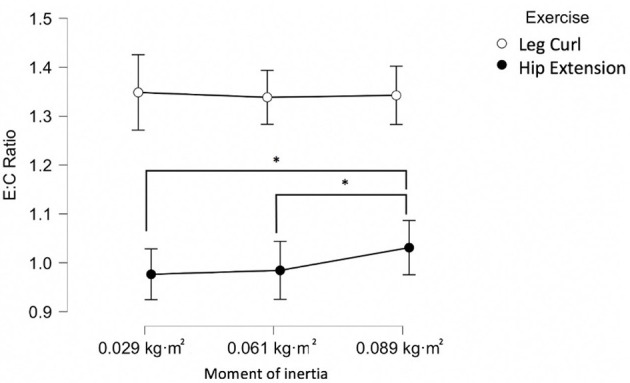
The eccentric:concentric (E:C) ratio for unilateral flywheel leg curl and hip extension exercises. *Statistically significant (*p* < 0.05) difference between E:C ratio.

Figure 4 highlights the larger eccentric peak power demands (represented as eccentric:concentric ratio) of the unilateral leg curl in comparison with the unilateral hip extension. Meanwhile, Table 2 reports *good* to *excellent* reliability for most concentric and eccentric measures and the large variations in reliability (*unacceptable to* good) of the eccentric:concentric ratio for both exercises.

**Table 2:** Unilateral flywheel leg curl and hip extension between session reliability.

**Moment of** **inertia and** **exercises**	**Concentric** **peak power** **ICC (95% CI)**	***Interpretation***	**Eccentric** **peak power** **ICC (95% CI)**	***Interpretation***	**Eccentric:concentric** **ratio peak power** **ICC (95% CI)**	***Interpretation***
0.029 kg·m^2^						
Hip extension	0.94 (0.85–0.97)	*Excellent*	0.95 (0.88–0.98)	*Excellent*	0.29 (−0.78; 0.72)	*Unacceptable*
Leg curl	0.85 (0.63–0.94)	*Good*	0.85 (0.64–0.94)	*Good*	0.89 (0.72; 0.96)	*Good*
0.061 kg·m^2^						
Hip extension	0.96 (0.86–0.99)	*Excellent*	0.96 (0.76–0.99)	*Excellent*	0.64 (0.10; 0.86)	*Questionable*
Leg curl	0.81 (0.48–0.93)	*Good*	0.74 (0.31–0.90)	*Acceptable*	0.77 (0.42; 0.91)	*Acceptable*
0.089 kg·m^2^						
Hip extension	0.97 (0.87–0.99)	*Excellent*	0.97 (0.88–0.99)	*Excellent*	0.54 (−0.17; 0.82)	*Poor*
Leg curl	0.88 (0.69–0.95)	*Good*	0.83 (0.59–0.93)	*Good*	0.85 (0.61; 0.94)	*Good*

*CON, Concentric peak power; ECC, eccentric peak power; E:C, eccentric:concentric peak power ratio; ICC, intra-class correlations; CI, confidence intervals*.

## Discussion

The aims of this study were to investigate how exercise parameters (concentric and eccentric peak power, eccentric:concentric ratio) were affected by alteration of flywheel moment of inertia (0.029–0.089 kg·m^2^) during different unilateral hamstring exercises and to determine their inter-session reliability. Leg curl reliability scores ranged from *acceptable* to *good*, while all hip extension reliability scores for concentric and eccentric peak power were rated as *excellent*. The eccentric:concentric peak power ratio was rated from *acceptable* to *good* and from *unacceptable* to *questionable* for leg curl and hip extension, respectively (Table 2). In disagreement with the hypothesis, greater peak power values were obtained with higher rather than lower moments of inertia during the hip extension exercise while no differences in peak power were seen between moments of inertia during the leg curl exercise. Greater eccentric:concentric ratio was reported with the highest moment of inertia during the hip extension exercise which supports the hypothesis that higher moments of inertia would obtain greater eccentric overload (Figure 3), while all moments of inertia achieved similar eccentric overload during unilateral leg curl (Figure 2).

Flywheel leg curl exercises have been effectively implemented in elite soccer, but the literature has focused predominantly on bilateral application (Askling et al., 2003; Sabido et al., 2018; Piqueras-Sanchiz et al., 2019, 2020). The present investigation is the first to suggest that peak power (concentric and eccentric) during the unilateral leg curl does not differ significantly between a range of moments of inertia (0.029–0.089 kg·m^2^) (Figure 2). The force-velocity-power relationships theoretically suggest that maximal power occurs at an intermediate resistance (Hill, 1938), although some investigations into the moment of inertia-power relationship during flywheel training do not support such a theory (Martinez-Aranda and Fernandez-Gonzalo, 2017; Sabido et al., 2018; Piqueras-Sanchiz et al., 2020). Specifically, the majority of previous findings report the highest peak power outputs at the lowest moments of inertia utilized (that are typically prescribed and applicable) (Tous-Fajardo et al., 2006; Martinez-Aranda and Fernandez-Gonzalo, 2017; Sabido et al., 2018; Piqueras-Sanchiz et al., 2020; O'Brien et al., 2021), while others and the present investigation report no differences in peak power between moments of inertia (Piqueras-Sanchiz et al., 2019; McErlain-Naylor and Beato, 2020). The present findings suggest that relationships between moments of inertia and peak power may be affected by several factors (i.e., exercise and participant) and therefore cannot be generalized. When attempting to quantify the effects of different moments of inertia and differences between individuals, it is worth considering that these effects may differ between measures (velocity, force, and peak power) (Muñoz-López et al., 2021a). Considering the unclear moment of inertia-power relationship presently reported at the group level in this study, it is recommended that practitioners utilize peak power with caution to determine which moment of inertia to utilize (as a measure of exercise intensity) during the unilateral leg curl exercise.

The present investigation reports that eccentric peak power is significantly greater than concentric peak power during the unilateral leg curl (Figure 4). In agreement with the present findings, bilateral flywheel leg curl exercises are able to achieve high eccentric peak power outputs using a variety of moments of inertia (Tous-Fajardo et al., 2006; Piqueras-Sanchiz et al., 2020; Pedersen et al., 2021). The present study supports the need to confirm eccentric overload with specific parameters as it is not always obtained (Muñoz-López et al., 2021a). Attaining high eccentric outputs during flywheel training is not only important to surveyed elite soccer practitioners but also appears important for improving eccentric strength and inducing morphological adaptations with athletic populations (Presland et al., 2020; Allen et al., 2021; de Keijzer et al., 2022). For example, bilateral flywheel hamstring leg curls did not obtain morphological nor strength improvements, whereas eccentric-biased flywheel leg curls improved fascicle length and strength (Presland et al., 2020). The present study suggests that unilateral flywheel leg curls are an effective and reliable method for obtaining eccentric overload and may therefore be effective for inducing desirable morphological and strength adaptations with athletic populations.

In contrast to the present findings, other investigations reported that no eccentric overload was obtained when using a unilateral (Suarez-Arrones et al., 2020) or bilateral flywheel leg curl (Tous-Fajardo et al., 2006). Although the moment of inertia (0.072 kg·m^2^) used previously in unilateral protocols is within the range of the present investigation (0.029–0.089 kg·m^2^), other factors may have influenced the outcomes between investigations. Specifically, the participants' flywheel training experience, the device characteristics and technique utilized are important to consider (Muñoz-López et al., 2021a). For example, considering the need for a maximal and prolonged familiarization to optimize eccentric peak power outputs (Sabido et al., 2018; Piqueras-Sanchiz et al., 2020), the submaximal familiarization (2 sets of 6–8 submaximal repetitions) previously utilized may have influenced eccentric peak power obtained (Suarez-Arrones et al., 2020). The present investigation reports that an eccentric overload of peak power may be achieved regardless of moment of inertia utilized with unilateral flywheel leg curl after sufficient familiarization (Figure 4). It is also possible that although there are no significant differences in eccentric overload attained between moments of inertia at the group level, such changes may occur for some individuals. Therefore, if practitioners wish to optimize training prescription, it would be ideal to verify if there are optimal moments of inertia to develop peak power with their athletes (Piqueras-Sanchiz et al., 2020; Suarez-Arrones et al., 2020).

Only the highest moments of inertia obtained a greater eccentric peak power relative to the concentric output during the flywheel hip extension exercise (Figure 3). In contrast to the present findings, altering moment of inertia (0.075–0.100 kg·m^2^) did not obtain eccentric overload during unilateral hip extensions with healthy young males (Piqueras-Sanchiz et al., 2019). Considering similarity in protocols, it is possible that study limitations (limited sample size, study design) biased the results and impacted conclusions (Piqueras-Sanchiz et al., 2019). The present findings support previous literature suggesting that higher moments of inertia elicit higher eccentric overload of peak power during flywheel training (Beato et al., 2021; Muñoz-López et al., 2021a). Nonetheless, blindly increasing moments of inertia may not significantly improve eccentric overload obtained—warranting objective measures of outputs (O'Brien et al., 2021). For example, during flywheel Romanian deadlifts, no significant difference was found between the eccentric overload achieved with a variety of higher inertias (0.050–0.100 kg·m^2^) (Suarez-Arrones et al., 2020). It is also recommended that practitioners be mindful of how altering moments of inertia may impact other factors (rate of perceived exertion, movement velocity) as well as peak power measures (Piqueras-Sanchiz et al., 2019). Analyzing how moments of inertia impact individual outputs rather than group outputs may also enhance understanding and utilization of moment of inertia-power relationships (O'Brien et al., 2021).

The unilateral flywheel hip extension has been investigated less than the leg curl exercise, however it is often utilized by practitioners to strengthen the hamstrings (Tous-Fajardo et al., 2016; Suarez-Arrones et al., 2020) and is considered a useful and practical exercise for reducing likelihood of muscular injuries to the hamstrings (Beato et al., 2021). The present findings show that the greatest moments of inertia obtained greater concentric (*small* to *moderate*; *d* = 0.53–0.95) and greater eccentric (*moderate* to *large*; d = 0.85–1.27) peak power outputs during unilateral flywheel hip extension than with lesser moments of inertia (Table 1). In contrast to the present findings, lower moments of inertia (0.025–0.050 kg·m^2^) obtained greater peak power when compared to higher moments of inertia (0.075–0.100 kg·m^2^) during flywheel Romanian deadlifts (O'Brien et al., 2021). The moment of inertia-power relationship between the two exercises may differ due to the biomechanical differences of the exercises (supine unilateral open kinetic chain vs. standing bilateral closed kinetic chain) (O'Brien et al., 2021). The present and aforementioned findings therefore support the theory that different exercises (considering equipment and execution) may alter moment of inertia-power relationships (Martinez-Aranda and Fernandez-Gonzalo, 2017; Sabido et al., 2018; Piqueras-Sanchiz et al., 2019; McErlain-Naylor and Beato, 2020; Worcester et al., 2020). In contrast to the present findings, previous investigation into unilateral hip extensions suggest that altering moments of inertia (0.075–0.100 kg·m^2^) did not impact peak power (Piqueras-Sanchiz et al., 2019). Differences in flywheel device characteristics utilized in the two protocols (vertical vs. horizontal cylindrical shafts varying in radius and disc diameter) (Muñoz-López et al., 2021a) as well as the different participant characteristics may partly explain disagreement between investigations. Although peak power provides a singular discrete value with no kinetic or kinematic information regarding the rest of the movement, the present investigation suggests that peak power measures can still be utilized as a basic method for prescribing exercise intensity during unilateral flywheel hip extensions at the group level. An alternative to monitoring peak power is the use of velocity measures (Muñoz-López et al., 2021a), with literature suggesting that velocity may be a more appropriate measure to quantify changes in moments of inertia. Nonetheless, further investigation into the prescription of flywheel training intensity is needed (Carroll et al., 2019; McErlain-Naylor and Beato, 2020).

Although concentric and eccentric peak power have become frequently applied as measures of intensity during flywheel training (Maroto-Izquierdo et al., 2021; Muñoz-López et al., 2021a), such measures do not summarize the intensity of the eccentric component in comparison to the concentric component (Nuñez et al., 2019). The eccentric:concentric ratio has become a practical method to monitor the demands of flywheel exercise in a single measure (Nuñez et al., 2019). Although ratios have been deemed to be problematic and easily misused (Curran-Everett, 2013), the eccentric:concentric ratio is still commonly researched and utilized (Muñoz-López et al., 2021a). Previously, participants who were experienced with bilateral flywheel leg curl attained higher eccentric:concentric ratios than inexperienced participants which suggests a sensitivity to flywheel resistance training experience (Tous-Fajardo et al., 2006). In agreement with previous literature, when inexperienced participants performed bilateral leg curl, the eccentric:concentric ratio obtained was not reliable (Piqueras-Sanchiz et al., 2020). Although applied and discussed frequently within the literature (Muñoz-López et al., 2021a), the present and previous investigations highlight that it currently remains unclear whether the eccentric:concentric ratio can be a reliable and effective tool for monitoring flywheel training (Maroto-Izquierdo et al., 2021). Overall and until further investigation, the eccentric:concentric ratio should be considered an unreliable parameter and should not be utilized (Piqueras-Sanchiz et al., 2020; Maroto-Izquierdo et al., 2021). Instead, practitioners should continue to use concentric and eccentric peak power parameters as reliable parameters for monitoring flywheel training (Table 2).

This study has several limitations—firstly, the sample enrolled (amateur male soccer players) may not represent how other populations (e.g., female or elite male soccer players) respond to changes in moments of inertia. Secondly, the group level analysis performed may have masked differences at the individual level, with future investigations potentially warranting individual analyses of responses to changes in moments of inertia. Finally, this study did not investigate the moment of inertia-velocity relationship. Future research could evaluate if velocity can be used to prescribe appropriate moments of inertia.

## Conclusions

This study reports that leg curl reliability ranged from *acceptable* to *good*, while all hip extension reliability scores for concentric and eccentric peak power were rated as *excellent*—highlighting that such parameters can be used by practitioners. The eccentric:concentric ratio should not be utilized for monitoring training outcomes. The present study is the first to determine that moments of inertia impact training parameters differently (concentric and eccentric peak power) between two unilateral flywheel hamstring exercises with the same population. Greater peak power values were obtained with higher rather than lower moments of inertia during the hip extension exercise while similar peak power measures were seen between moments of inertia during the leg curl exercise. The moment of inertia-power relationship between the two exercises may differ due to biomechanical differences between the exercises. Accordingly, such differences must be considered by practitioners when periodizing and planning training, possibly warranting individualization of moment of inertia to optimize peak power.

## Practical Applications

The present investigation provides novel insight and recommendations for the prescription of flywheel training. Specifically, a variety of moments of inertia (0.029, 0.061, and 0.089 kg·m^2^) can be prescribed during unilateral flywheel leg curl to achieve high eccentric knee flexor demands. With regards to unilateral hip extension, practitioners are recommended to utilize higher moments of inertia (e.g., 0.089 kg·m^2^) to obtain greater eccentric overload, as well as higher individual concentric and eccentric peak power outputs. Different exercises may have different moment of inertia-power relationships because of the biomechanical differences between exercises—warranting careful consideration for training prescription. Although this study demonstrates that peak power can be a useful parameter to use in daily practice, practitioners may also wish to measure other parameters (e.g., velocity) to further characterize training demands and outcomes. Overall, practitioners must consider that obtaining high peak power outputs are not the sole objective in physical preparation, whereby many different considerations must be made to optimize performance (e.g., focus on high velocity movements with lower moments of inertia). If practitioners cannot obtain individual moment of inertia-power relationships or monitor training load objectively with a group of athletes, the present findings can be used to guide basic flywheel hamstring training prescription. Finally, this study suggests avoiding the use of the eccentric:concentric ratio as a parameter with flywheel training.

## Data Availability Statement

The raw data supporting the conclusions of this article will be made available by the authors, without undue reservation.

## Ethics Statement

The studies involving human participants were reviewed and approved by Ethics Committee of the University of Suffolk. The patients/participants provided their written informed consent to participate in this study.

## Author Contributions

All authors listed have made a substantial, direct, and intellectual contribution to the work and approved it for publication.

## Conflict of Interest

The authors declare that the research was conducted in the absence of any commercial or financial relationships that could be construed as a potential conflict of interest.

## Publisher's Note

All claims expressed in this article are solely those of the authors and do not necessarily represent those of their affiliated organizations, or those of the publisher, the editors and the reviewers. Any product that may be evaluated in this article, or claim that may be made by its manufacturer, is not guaranteed or endorsed by the publisher.
