# Supplementary figures and images for: The Effect of Flywheel Inertia on Peak Power and Its Inter-session Reliability During Two Unilateral Hamstring Exercises: Leg Curl and Hip Extension
Source: Front Sports Act Living. 2022 Jun 10;4:898649. doi: 10.3389/fspor.2022.898649 (PMC9226424; doi:10.3389/fspor.2022.898649)

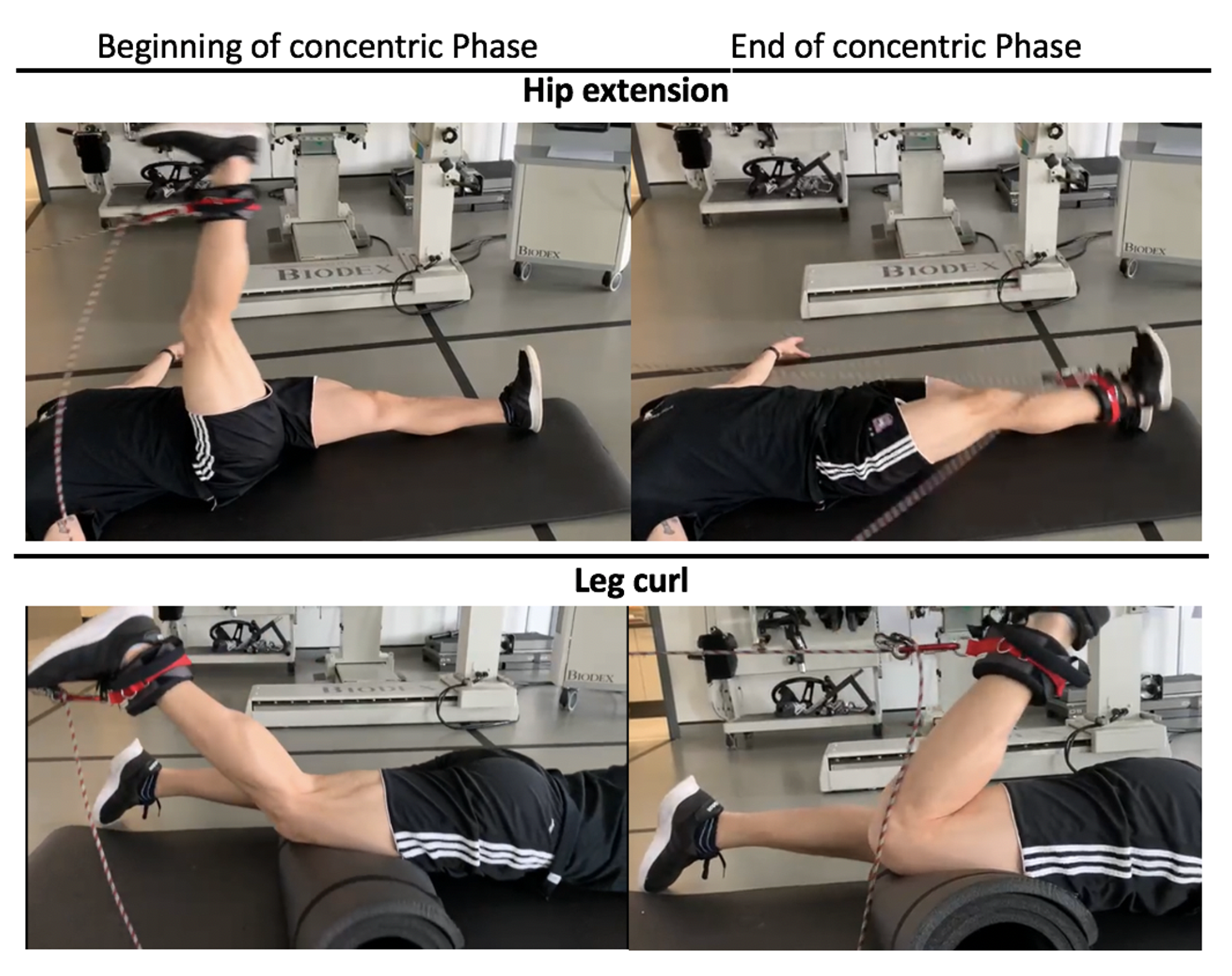

Supplement: Supplementary file 1 [file Image_1.PNG]

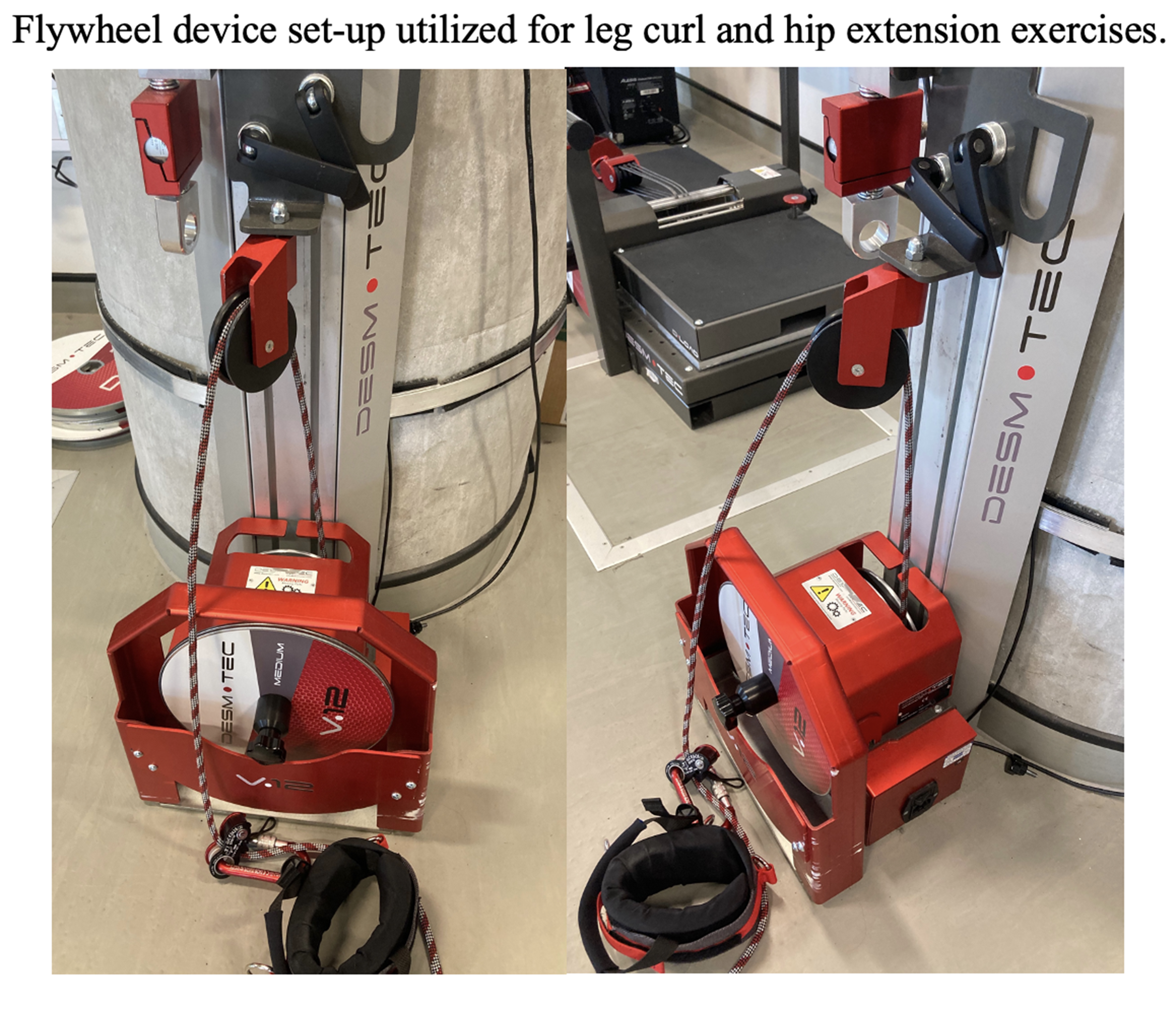

Supplement: Supplementary file 2 [file Image_2.PNG]
